# Define, process and describe the intersectoral embedded carbon flow network in China

**DOI:** 10.1016/j.mex.2019.08.003

**Published:** 2019-09-03

**Authors:** Kaiyao Wu, Tinggan Yang, Xiaoyan Wei

**Affiliations:** a School of Statistics and Information, Shanghai University of International Business and Economics, Shanghai 201620, China; b Shanghai Key Laboratory of Financial Information Technology, Shanghai 200433, China; c School of Statistics and Mathematics, Shanghai Lixin University of Accounting and Finance, Shanghai 201209, China; d School of Accounting, Shanghai Lixin University of Accounting and Finance, Shanghai 201209, China

**Keywords:** Input–output, Carbon intensity, Life cycle assessment, Social network analysis

## Abstract

This article focuses on defining the intersectoral embedded carbon flow network as a matrix to mimic the complex economic-energy-environment symbiotic system in China. We propose a set of synthetical methodologies, which combines life cycle assessment (LCA) and social network analysis (SNA) in the input–output framework. The nodes and relations between nodes in the network are delicately designed such that these relations, which represent the carbon intensity of total intersectoral input between sectors, can be comparable among sectors and over time. Subsequently, based on longitudinal data of input–output tables in China, we derive, sequentialize and dichotomize matrices in order to apply the SNA method to describe the evolution of the intersectoral embedded carbon flow network. The SNA methods used include network visualization, triad census, cohesion metrics, position metrics and core–periphery modeling. Our synthetical methodologies provide a potential systematic solution to carbon reduction in China and help policy makers determine policy priorities rationally.

• By constructing an intersectoral embedded carbon flow network matrix, we provide an easily explicable map to aid in the investigation and research in human derived CO_2_ emissions embedded in the network.
• By describing the longitudinal network matrices with SNA, the evolution of the complex economic-energy-environment symbiotic system in China can be mapped out, such as an example illustrated in Wu et al. [1].

## Graphical abstract

**Figure:**
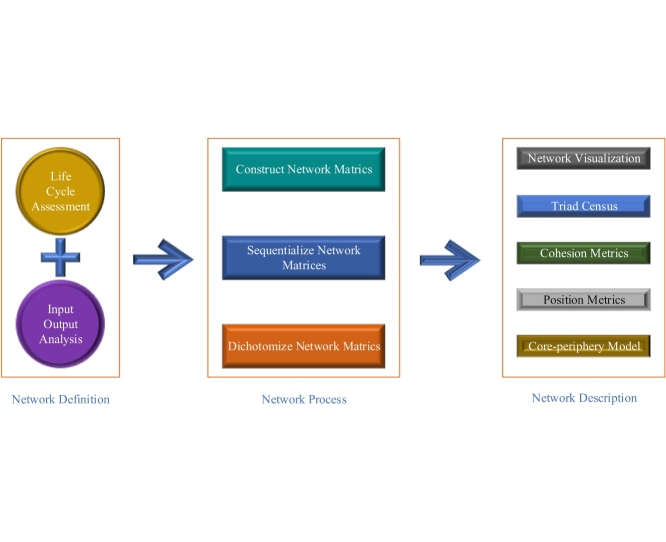

**Specifications Table**

**Table:** 

Subject area:	*Environmental Science*
More specific subject area:	*Greenhouse gas emission reduction*
Method name:	*Life cycle assessment and social network analysis*
Name and reference of original method:	*R.E. Miller, P.D. Blair, Input–output analysis: foundations and extensions [M]. Cambridge University Press, 2009.*https://ideas.repec.org/b/cup/cbooks/9780521517133.html
	*Y. Yang, R. Heijungs, M. Brandão, Hybrid life cycle assessment (LCA) does not necessarily yield more accurate results than process-based LCA. J. Clean. Prod. 150 (2017) 237–242.*https://www.sciencedirect.com/science/article/pii/S0959652617304407
	*S.P. Borgatti, M.G. Everett, J.C. Johnson, Analyzing social networks [M], 2018. Sage.*https://journals.openedition.org/lectures/24709
Resource availability:	*A set of synthetical methodologies, which combines life cycle assessment (LCA) and social network analysis (SNA) in the input–output framework.*

## Method details

It is useful to represent a complex economic-energy-environment symbiotic system as a network where the sectors (usually denoted as “nodes” in social network analysis (SNA) literatures) represent the components, and the links among the sectors represent their interactions [2]. Since sectors are an economic component of the whole complex system which consumes energy and produces carbon emissions, intersectoral carbon flow embedded in the supply chain is correspondingly regarded as an interactive link among sectors. Since it is the sectors and the interaction among sectors which give rise to the collective behaviors of a network system, they must be given careful consideration if we are to address the many important questions, such as interactive pattern and network dynamic, regarding a system with this degree of complexity [3].

The input–output model, initially proposed by Leontief [4] and further developed by many researchers [5], describes the interaction between economic sectors, and was extended with environmental information in order to demonstrate the interaction between the economy and the environment in the complex economic-energy-environment symbiotic system. However, most literatures only use input–output models to derive metrics measuring sectoral or national behavior [6], [7], [8], such as carbon intensity [9]; the abundant source of information related to network structure in the input–output models, especially interaction and network between sectors, was largely ignored, leading to only an obscure understanding of the system.

We attempt to imitate the complex economic-energy-environment system in China through an input–output (IO) based intersectoral embedded CO_2_ emission flow network, which consists of employing both life cycle assessment (LCA) and social network analysis (SNA) in order to derive valuable insights into carbon emissions embedded in the complex symbiotic system network.

### Network definition

Hereafter, using the input–output framework, we develop a general network pattern to mimic the economic-energy-environment symbiotic system, in the form of a matrix, based on the methodologies of LCA and SNA. It should be pointed out that China has had a tendency to shift gradually from an investment to a consumption-driven economy, and production structures and consumption patterns are likely to be the main driving forces in the reduction of emissions in China in the future [10]. This paper focuses on consumption carbon footprint, which can be regarded as the derivative of these two driving forces based on consumption-principle accounting.

Firstly, we assume a vector of emission coefficients, *c* = [*c*_*j*_], each element of which is the amount of emission generated per unit of sector *j*’s output. Extending this we state that *c*_*j*_ = *C*_*j*_/*X*_*j*_, where *C*_*j*_ is the emission from sector *j* based on the production principle of emission accounting (which is referred to as energy related CO_2_ emission in this paper) and *X*_*j*_ is sector *j*’s total output.

We denote *A* as a non-negative matrix with direct input coefficient *a*_*ij*_, where *a*_*ij*_ ≥ 0 for all *i* and *j*. The sum of the elements in the *j*th column of *A* indicates inputs supplied from other sectors that are used in making a unit of final product for sector *j*. We denote *f* as the vector of total final consumption. LCA should take into account the entire supply chain for a product, including indirect suppliers (supplier to supplier). Allowing for added levels of supplier interactions, the total supply of a final product from the various stages can be calculated as: $X=(I+A+A^{2}+A^{3}+\cdots )\times f$. This exactly represents the traditional Leontief model *X* = *L* × *f*, where *L* can be denoted as *L* = (*I* − *A*)^−1^, a matrix with total input coefficient *L*_*ij*_, which is the total supply of *j* required to produce a unit of *i*. This approach arises directly from the basic assumption of the input–output model that the output is demand generated through final demand [5].

We can compute the total carbon footprint generated by the economy directly and indirectly supporting final consumption by extending the traditional Leontief model like so: *TC* = *c* × *L* × *f*.

With further modifications, we can get an intersectoral embedded carbon flow matrix:

(1) $$C=\hat{c}\times L\times \hat{f}$$

where $\hat{c}$, $\hat{f}$ are the diagonalized vectors of emission coefficients and final consumption, respectively. The typical element of this matrix, *C*_*ij*_, measures the amount of emission flow from sector *i* to *j*, which is embodied in the supply network required by final consumption in sector *j*, directly and indirectly.

According to the manner in which it is defined, the matrix is a function of three parts, representing three symbiotic sub-systems in the real world, where $\hat{c}$ is a proxy for the system of technology, *L* is a proxy for the production system, and $\hat{f}$ is a proxy for the system of final consumption. These three sub-systems interact and interweave together to form a dynamic intersectoral embedded carbon flow network. The matrix reclassifies emissions from ‘pure sectors’ into ‘vertically integrated sectors’ based on the final consumption of an economy, according to the input–output framework. Simultaneously, the aggregate carbon footprint is divided into intersectoral carbon flows, which are embedded in corresponding supply flows. Accordingly, the matrix provides a road map to reducing carbon emissions from the perspective of the supply chain.

The matrix expression described in formula (1) lays a foundation for SNA as it denotes a straightforward framework for the network. However, it is best to explicitly define the nodes and relations before we go further.

There are few issues in defining sectors as nodes, except that the sector classification should be kept consistent in longitudinal networks, which is a prerequisite to having economic and environmental data align with each other. The absolute emission flow between inter-sectors denoted by every element of the matrix can effectively be regarded as “relations”. However, proper ‘relative’ intersectoral emission flows should be chosen in order to get rid of the scale problem, so that network analysis can be applied to the specific research objective. In actuality, network intersectoral analysis is quite sensitive to this; if we make no adjustment at all, the intersectoral embedded emission would show higher values in latter years than in former years. This is due to the fact that scale of emission always increases as the volume of the economy increases, but the fact is that intersectoral embedded emission bonding is less connected over time.

An alternative option is to descale *C* in formula (1) to derive: $CI=\hat{c}\times L$ The typical element in matrix *CI*_*ij*_ measures the intensity of environmental repercussions according to the supply interaction between sector *i* and *j* from the context of LCA. It is flexible due to descaling, and is comparable among sectors and over time because of consistency. *CI* also has a shortcoming, however, which is that it ignores the economic structure; the consequence of which is that sectors with high intensity of environmental repercussions but that are trivial in size will be emphasized. By adding sectoral proportions of final consumption as weights to formula (2), we get:

(2) $$ACI=\hat{c}\times L\times \hat{str}$$

where $\hat{str}$ is a diagonalized vector of sectoral proportions of final consumption (hereafter denoted as *str*_*i*_, for sector *i*). *ACI* gets rid of the scale of the economic aggregate, retaining the economic structure of final consumption, and focuses the analysis on the whole national network. As such, *ACI* provides an easily explicable map through which to investigate intersectoral embedded CO_2_ emission flow from the perspective of the network.

*ACI* contains three coefficients matrices, namely emission coefficients, total intermediated coefficients and economic structure coefficients, the latter two being independent from commodity prices. In order to have comparable longitudinal data for $\hat{c}$, price adjustment of emission coefficients should be considered. With this in mind, *ACI*_*ij*_, denoting “carbon intensity of total intersectoral input from sector *i* to sector *j*”, is defined as the “relation” between “nodes”, and is comparable among sectors and over time.

The sum of the elements in the *i*th row of *ACI*, denoted as *ACI*_*i*_, indicates sector *i*'s carbon intensity as a supplier based on IO-LCA. This is shown in formula (3):

(3) $$ACI_{i}=\sum_{j}ACI_{ij}$$

The sum of all elements in *ACI*, denoted as *TACI*, indicates carbon intensity of national consumption based on IO-LCA. This is shown in formula (4):

(4) $$\mathrm{TACI}=\sum_{ij}ACI_{ij}$$

*ACI*_*i*_ and *TACI* appear to represent metrics of sectoral and integral behavior contained in our intersectoral embedded carbon flow network structure.

### Network process

The subsequent step is to derive a time series of matrices for Chinese *ACI* and related variables of sectoral attribution, such as *ACI*_*i*_, *f*_*i*_, *str*_*i*_, as well as carbon intensity of national consumption *TCI*. Datasets should be derived for each factor in the right hand of formula (1). Essentially, most factors can be extrapolated from input–output tables, except $\hat{f}$ which should be extrapolated from input–output tables and carbon emission accounting. Unfortunately, we cannot obtain detailed original emission data for sectors, so we focused instead on CO_2_ emissions from energy use, which account for the majority of greenhouse gas emissions. Full-blown methodology to calculate emissions from energy consumed has already been developed, thus what we need is energy statistics, which has a solid data foundation in China.

#### Sectors reclassified

With consideration for consistency in industry classification, we chose input–output tables from China, including “final energy consumption by industrial sector (standard quantity)” and energy balance of China (standard quantity)”, sourced from the China Energy Statistical Yearbook (for the years of 1997, 2002, 2007 and 2012). The input–output tables were traditionally constructed based on assumptions of domestic technology, though the latter two were used to derive the matrix of terminal energy products consumed in every sector and to extend the traditional input–output tables.

In accordance with corresponding relationships between large categories, small categories and subcategories in the National Economic Industry Classification Standard (GB/T 4754-2011) which were used in input–output accounting and energy statistics by the National Bureau of Statistics of China, the sectors were merged and adjusted to 28 sectors, as shown in Table 1. The input–output tables and the matrix of terminal energy products consumed were then aggregated and manipulated into the structures of 28 sectors.

**Table 1:** Abbreviations of sector names. Industry classifications are readjusted into 28 sectors in this paper. This table lists the names and abbreviations for these readjusted sectors.

Abbreviation	Sector name
agr	Agriculture, hunting, forestry and fishing
coa	Coal mining
oil	Oil and natural gas industry
met	Metallic mining and quarrying
nme	Non-metallic mining and quarrying
foo	Food products, beverages and tobacco
tex	Textile
clo	Apparel, leather, feather, other fiber products
woo	Wood and products of wood and cork
pap	Pulp, paper, paper products, printing and publishing
pet	Refined petroleum products, coke, and nuclear fuel
che	Chemicals, chemical products, rubber and plastics products
nmm	Other non-metallic mineral products
mes	Metal smelting and rolling processed product
fab	Fabricated metal products,
mac	Machinery and equipment n.e.c
tra	Transport Equipment
ema	Electrical machinery and apparatus n.e.c
ete	Radio, television and communication equipment
ins	Office, accounting and computing machinery
scr	Manufacturing n.e.c; recycling
pow	Steam hot water and electricity supply
gas	Gas supply
tap	Water supply
bui	Construction
trs	Transportation, storage and communications
who	Wholesale and retail trade and catering
oth	Other service industries
eco	Whole economy

#### Sectoral CO_2_ emission

A variety of energy consumption can lead to CO_2_ emissions, including combustion emissions and fugitive emissions. Since fugitive emissions are relatively small, we only calculate combustion emissions in this paper. According to the IPCC national GHG inventory guidelines, total CO_2_ emissions can be calculated by estimating CO_2_ emissions from various energy sources and totaling them to retrieve the aggregate amount.

The equation for CO_2_ emissions caused by energy combustion is as follows:

(5) $$C=\sum_{i}EC_{i}\times ef_{i}\times (1-cs_{i})\times o_{i}\times \left(\frac{44}{22}\right)$$

where *C* is carbon dioxide emissions, *i* is energy product type, *EC* is total energy consumption, *ef* is emission factor of the energy type, *cs* is proportion of energy which is not oxidized but is retained as raw material in the product, *o* is oxidation fraction of carbon, and constants 44 and 12 are modal weights for CO_2_ and C, respectively. In order to simplify the calculation, *cs* is set to 0 and *o* is set to 1, since these are close approximations to the actual scenario. The energy products consumed in every sector involve 30 different types, whose emission factors for CO_2_ are shown in Table 2.

**Table 2:** CO_2_ emission factors of energy products. These emission factors of certain energy types are estimated according to the guidelines given by IPCC.

Raw Coal	2.76299	Fuel Oil	2.26782
Cleaned	2.77178	Naphtha	2.14769
Washed	2.77178	Lubricants	2.14769
Briquettes	2.81573	Paraffin	2.14769
Gangue	2.76299	White Spirit	2.14769
Coke	3.1351	Bitumen	2.14769
Coke Oven	1.30092	Petroleum	2.14769
Blast Furnace	1.30092	LPG	1.84883
Converter	1.30092	Refinery	1.68768
Other Gas	5.9186	Petroleum	2.14769
Coking	2.85675	Natural Gas	1.64373
Crude Oil	2.14769	LNG	1.64373
Gasoline	2.051	Heat	2.77178
Kerosene	2.09495	Electricity	2.24237
Diesel Oil	2.14769	Other	2.93

#### Sequentialized matrices

To derive comparable longitudinal data for $\hat{f}$, the price adjustment approach as detailed by the China Statistical Yearbook of 2013 was followed, where constant price GDP and current price GDP were used to calculate rate of inflation for 1997, 2002, 2007 and 2012 based on prices from 2010 (which are 1.491, 1.4549, 1.1422 and 0.9095, respectively). The values in relativized vector $\hat{f}$ for the 4 years (based on the producer current price at the accounting year) are divided by their respective inflationary rate, after which we can get time comparable $\hat{f}\text{s}$, based on constant prices from 2010.

With the data prepared as described above, we have worked out sequentialized *ACI*s for 1997, 2002, 2007 and 2012, applying formula (2).

#### Dichotomized matrices

In order to highlight the network structure, we dichotomized each wave of *ACI* to retrieve *CACI*, using the following dichotomization rule: if *ACI*_*ij*_ is greater than 0.005 then *CACI*_*ij*_ = 1, otherwise *CACI*_*ij*_ = 0, where the diagonal elements of the output matrix follow the dichotomization rule. The same dichotomization rule is applied consistently over time so that the transformed matrices have comparable basis, resulting in intersectoral networks of ‘strong’ carbon flows. The frequency of *CACI* values of 1 were 50, 31, 32, 23 in four respective waves (13, 9, 8, 8 in diagonal positions), which consist of the strongest carbon flows embedded in corresponding supply chains. Less than 10% of the total flows in all four waves were regarded as links in the dichotomized matrices.

### Network description

#### Visualization with MDS

We used “iterative metric multi-dimensional scaling (MDS)^1^” in NetDraw^2^ to generate coordinates based on similarity, adjusted to the nearest Euclidean. Since our intent was primarily to explore the network, no changes were made to options regarding node attribute appearance, maintaining the default neat style.

#### Triad census

In the triad census, dyad configurations were monitored. As shown in Table 3, 16 possible triadic configurations of directed non-reflexive graphs can be counted. Type 003 is a combination of 3 unconnected nodes, or 3 null dyads. Type 012 consists of asymmetric dyads, while Type 102 is made up of mutual dyads. Others include in-star, out-star, transitive and intransitive triads, all having no less than 2 links to bond 3 nodes.

**Table 3:** Triad census. 16 possible triadic configurations of directed non-reflexive graphs are counted in this triad census, which are further classified into 3 categories.

	1997	2002	2007	2012
**003/categoryI**	2290	2606	2617	2759
**012**	713	548	514	441
**102**	17	0	15	0
**categoryII**	730	548	529	441
**021D**	104	45	45	20
**021U**	118	61	54	48
**021C**	1	2	6	3
**111D**	1	0	7	0
**111U**	0	0	1	0
**030T**	24	14	14	5
**030C**	0	0	0	0
**201**	0	0	0	0
**120D**	8	0	3	0
**120U**	0	0	0	0
**120C**	0	0	0	0
**210**	0	0	0	0
**300**	0	0	0	0
**categoryIII**	256	122	130	76

*Note*: Dich Threshold 5% of 2002, gt 0.0082 and including diagonal, 1. 003 = A, B, C, the empty subgraph. 2. 012 = A->B, C, subgraph with a single directed edge. 3. 102 = A<->B, C, the subgraph with a mutual connection between two nodes. 4. 021D = A<-B->C, the out-star. 5. 021U = A->B<-C, the in-star. 6. 021C = A->B->C, directed line. 7. 111D = A<->B<-C. 8. 111U = A<->B->C. 9. 030T = A->B<-C, A->C. 10. 030C = A<-B<-C, A->C. 11. 201 = A<->B<->C. 12. 120D = A<-B->C, A<->C. 13. 120U = A->B<-C, A<->C. 14. 120C = A->B->C, A<->C. 15. 210 = A->B<->C, A<->C. 16. 300 = A<->B<->C, A<->C, complete subgraph. 9, 12, 13, 16 are transitive; 6, 7, 8, 10, 11, 14, 15 are intransitive.

#### Cohesion metrics characterizing the whole network

Using cohesion metrics measuring the whole network, we intended to measure how strongly or closely related nodes in a network are to each other according to the cohesion approach. As shown in Table 4, these five metrics are positive and based on “dyads”. The first four are metrics based on the cohesion approach, and as such we may call them pure cohesion metrics; the fifth measures the extent to which the whole network is dominated by a single node according to the position approach, in combination with cohesion characteristics based on the position approach (thus we may call it a cohesion metric blended with positional information). Their conception and formulas are demonstrated below. For this paper, their values were calculated with UCINET, ignoring reflexive links but not ignoring direction of links (if direction is applied in the formula).

(1) Avg Degree *AD*(*t*), 0≤AD(t)≤n−1, is the average degree in the underlying graph, which is the simplest metric of cohesion:
  (6) $$AD(t)=\frac{\sum_{i}\sum_{j(i\neq j)}d_{ij}(t)}{n}$$
  where degree *d*_*ij*_(*t*) is equal to 1 if a link exits between nodes *i* and *j* in period *t*, and is equal to 0 if no link exits.
(2) Density *δ*(*t*), with 0 ≤ *δ*(*t*) ≤ 1, is the number of links divided by the maximum number of possible links (elements on the diagonal are ignored):
  (7) $$(t)=\frac{\sum_{i}\sum_{j(i\neq j)}d_{ij}(t)}{n(n-1)}$$
(3) Connectedness *CN*(*t*) with 0≤CN(t)≤1, is defined as the proportion of pairs of nodes that can reach each other by a path of any length. It can be calculated by 1 minus the fragmentation, which is the proportion of pairs of nodes that are unreachable:
  (8) $$CN(t)=1-\frac{V}{n(n-1)}$$
  where *V* represents pairs of nodes that are unreachable.
(4) Compactness *CP*(*t*), with 0≤CP(t)≤1, is the distance based cohesion metric weighting the path connecting nodes inversely by their length. It has a value of 1 when the network is a clique (everyone is adjacent) and zero when the network is entirely made up of isolated nodes:
  (9) $$CP(t)=\frac{\sum_{\left(i\neq j\right)}\left[1/g_{ij}(t)\right]}{n(n-1)}$$
  where *g*_*ij*_(*t*) is the geodesic distance from *i* to *j*, and 1/*g*_*ij*_(*t*) is set to 0 when no path exists from *i* to *j*.
(5) Degree Centralization *C*(*t*), with 0≤C(t)≤n−1, is calculated based on the concept of degree centrality (see below) by applying equation [6], regardless of supply/demand directionality:
  (10) $$C(t)=\frac{\sum_{j}\left(C_{j}\times (t)-C_{j}(t)\right)}{{\left(n-1\right)}^{2}}$$

**Table 4:** Metrics of cohesion. The values of these metrics are derived with UNICET, according to the formulas in Section 3.2.

	1997	2002	2007	2012
**Avg Degree**	1.786	1.107	1.143	0.821
**Density**	0.066	0.041	0.042	0.030
**Connectedness**	0.069	0.042	0.060	0.034
**Compactness**	0.067	0.042	0.051	0.032
**Deg Centralization**	0.447	0.315	0.353	0.207

*Note*: Dich Threshold gt 0.01 and including diagonal.

#### Position metric: degree centrality

Cohesion metrics characterizing the whole network provide single numbers to describe relevant aspects of the network, but tell nothing about how nodes and links are distributed. Degree centrality^3^ is actually the measurement of a node's position in the whole network. The higher the value of degree centrality, the more important the position taken by the sector will be [11]. The row sums of the adjacency matrix were denoted as indegree: $DC_{i}^{\mathrm{in}}(t)=\sum_{j(j\neq i)}d_{ji}(t)$, with $0\leq DC_{i}^{in}(t)\leq n-1$, measured as the number of incoming ties received from others. The column sums were denoted as outdegree: $DC_{i}^{out}(t)=\sum_{j(j\neq i)}d_{ij}(t)$, with $0\leq DC_{i}^{out}(t)\leq n-1$, measured as the number of outcoming ties received by others. Summing indegree and outdegree, we get degree centrality *DC*_*i*_(*t*), with $0\leq DC_{i}(t)\leq 2(n-1)$.

(11) $$DC_{i}(t)=\sum_{j(j\neq i)}d_{ji}(t)+\sum_{j(j\neq i)}d_{ij}(t)$$

#### Core–periphery model

This model is used to partition nodes into two groups (core and periphery) based on their positional equivalents [12]. The fundamental idea underlying the notion of positional equivalence is that of structural correspondence or similarity. With the core–periphery model, we are able to include both the idea of relation between two core members and the idea of internal equivalent, as well as the idea of a separation from members outside the core. In the core–periphery pattern, we can observe 4 blocks in the class blocked adjacency matrix, namely the core, core–periphery, periphery–core and periphery. Since nodes that are position equivalent would exhibit similar behaviors and outcomes, the partitioning of core nodes and periphery nodes has some meaningful implication.

### Application example

In a recent research project [1], we use the method expounded in this paper to give carbon emissions in China a more systemic and explicit representation, based on deliberately manipulated longitudinal data from 1997, 2002, 2007 and 2012. The evolvement of the intersectoral embedded carbon flow network and the manner in which carbon emissions co-evolved with the flow network were extensively explored and analyzed (from integral/nation and node/sector levels respectively), using the SNA method of network visualization, triad censuses, cohesion metrics, position metrics and the core–periphery model. The results are meaningful to target setting and implementing plans for emission reduction. Policy makers in the field of emission reduction would do well to apply and make use of our method, since it represents a more comprehensive and systematic solution.

## Footnotes

1 This method is detailed in Chapter 4 of Hanneman, R.A., & Riddle, M. (2005). Introduction to social network methods. At: http://faculty.ucr.edu/∼hanneman/nettext/.

2 NetDraw is a network visualization tool in the UCINET Software.

3 Degree centrality has long been considered crucial to understanding a node's position in the whole network [11].
